# Older American Indians’ Perspectives on Health, Arthritis, and Physical Activity: Implications for Adapting Evidence-Based Interventions, Oregon, 2013

**DOI:** 10.5888/pcd13.160098

**Published:** 2016-06-23

**Authors:** Kathleen P. Conte, Marc B. Schure, R. Turner Goins

**Affiliations:** Author Affiliations: Marc B. Schure, Montana State University, Bozeman, Montana; R. Turner Goins, Western Carolina University, Cullowhee, North Carolina.

## Abstract

**Introduction:**

Despite the high prevalence of arthritis and physical disability among older American Indians, few evidence-based interventions that improve arthritis self-management via physical activity have been adapted for use in this population. The purpose of this study was to identify beliefs about health, arthritis, and physical activity among older American Indians living in a rural area in Oregon to help select and adapt an arthritis self-management program.

**Methods:**

In partnership with a tribal health program, we conducted surveys, a focus group, and individual interviews with older American Indians with arthritis. Our sample comprised 6 focus group participants and 18 interviewees. The 24 participants were aged 48 to 82 years, of whom 67% were women. Forms B and C of the Multidimensional Health Locus of Control (MHLC) instrument, modified for arthritis, measured MHLC.

**Results:**

The concepts of health, arthritis, and physical activity overlapped in that health was a holistic concept informed by cultural teachings that included living a healthy lifestyle, socializing, and being functionally independent. Arthritis inhibited health and healthy behaviors. Participants identified barriers such as unreliable transportation and recruiting challenges that would make existing interventions challenging to implement in this setting. The Doctor subscale had the highest MHLC (mean = 4.4 [standard deviation (SD), 1.0]), followed by the Internal subscale (3.9 [SD, 1.4]) and the Other People subscale (2.8 [SD, 1.1]).

**Conclusions:**

Existing evidence-based programs for arthritis should be adapted to address implementation factors, such as access to transportation, and incorporate cultural values that emphasize holistic wellness and social interconnectedness. Culturally sensitive programs that build on indigenous values and practices to promote active coping strategies for older American Indians with arthritis are needed.

## Introduction

American Indians have the highest prevalence of arthritis of any population in the United States (1). Although physical activity is considered a central component of nonpharmacologic arthritis management (2), American Indians are less likely to engage in physical activity than are people from other racial/ethnic groups (3). The Centers for Disease Control and Prevention (CDC) publishes a list of recommended evidence-based physical activity programs for arthritis (4). However, to our knowledge, no studies have described successful adaptations of these programs for use among American Indians, or in rural settings, where as many as 48% of older American Indians reside (5). These statistics illustrate the need for culturally tailored physical activity programs to manage arthritis for American Indians. For evidence-based programs to be effective, adaptations may be needed to ensure that programs align with the cultural values of the new target population and context of the new setting (6). Understanding lay health beliefs can provide insight into motivations that underlie individuals’ health behaviors and coping strategies and inform relevant adaptations to existing programs (7). The purpose of this study was to identify beliefs about health, arthritis, and physical activity among older American Indians to help select and adapt an arthritis self-management and physical activity program for a tribe in rural Oregon.

## Methods

In 2013, we collaborated with a federally recognized tribe’s health program in rural Oregon to interview older, tribal-dwelling adults about their meaning of health, experience of arthritis, and interest in a group-based physical activity program. The study protocol was approved by the Oregon State University institutional review board and the tribe’s Health Board and Tribal Council. Partners from the tribal health clinic and the study team recruited participants by word-of-mouth and by distributing flyers at local events and facilities. We used purposive sampling to target older adults with arthritis and ensure that men were sufficiently represented. Enrolled tribal members who had self-reported arthritis and identified as older adults were eligible to participate. The tribe did not want to exclude people who might benefit from a program for older adults, so people who identified as older adults self-selected into the study. Previous studies indicate that reservation-dwelling American Indians aged 45 years have health status similar to that of non-American Indians aged 65 years (8).

We scheduled several 90-minute focus groups at various times during February through September 2013. Only 6 people attended the first focus group, and subsequent groups were canceled because of insufficient registration. Therefore, we offered community members the option of participating in 30- to 45-minute in-depth face-to-face interviews. Our final sample of 24 participants included 6 focus group attendees and 18 interviewees. The focus group and interviews were audio-recorded and transcribed verbatim. Participants completed a self-administered survey and received $50 for their participation.

The self-administered survey collected data on age, sex, education level, and marital status. Health was measured with a self-reported 6-item response (range, 1 = very poor, 2 = poor, 3 = fair, 4 = good, 5 = very good, 6 = excellent) (9). Participants ranked arthritis interference with daily activities in the past 6 months on a 10-point scale where 0 indicated no interference and 10 indicated unable to carry on any activities (10). Locus of control, or the extent to which people believe their health is within their control, due to chance, or in the hands of others, is predictive of health behaviors (11), so we included 2 forms of the Multidimensional Health Locus of Control (MHLC) instrument in the questionnaire (12). We used the MHLC Form B to assess locus of control associated with general health and Form C to assess locus of control associated with arthritis. Form B, previously validated among American Indians (13,14), assesses general health locus of control via three 6-item subscales (Internal, Powerful Others, and Chance). The condition-specific Form C, modified for arthritis, subdivides the Powerful Others scale into two 3-item subscales: Doctors and Other People.

In collaboration with tribal partners, we developed a semistructured guide adapted for both the focus group and interviews. The open-ended questions reflected 4 domains: the participants’ definition of health, effect of arthritis on peoples’ lives, barriers to physical activities, and preferences for a physical activity program.

Using Stata v12 (StataCorp LP) for the self-administered survey data, we obtained the mean scores for health variables and for each MHLC subscale. Cronbach’s ɑ was acceptable for Form B (range, α = 0.65–0.83) and Form C (α = 0.77–0.87) except for the Other People subscale (α = 0.37; Table 1). Alpha levels for the MHLC typically range from 0.60 to 0.75 (12,13).

**Table 1:** Mean Scores of Arthritis-Specific and General Domains of Health Locus of Control of Older Adult American Indians With Arthritis, Multidimensional Health Locus of Control Instrument, Oregon, 2013

Domain	Cronbach’s α	Mean (Standard Deviation)
Arthritis-specific^a^		
Doctors	.82	4.4 (1.0)
Internal	.87	3.9 (1.4)
Other people	.37	2.8 (1.1)
Chance	.77	2.6 (1.1)
**General health**		
Internal	.83	4.4 (1.2)
Powerful others	.71	3.5 (1.1)
Chance	.65	2.7 (1.0)

a Mean score range, 1–6; higher scores indicate attributing greater control to respective domain.

For the qualitative data, we used inductive content analysis to analyze the focus group and in-depth interview data (15). Initially, the 3 researchers separately reviewed and coded a transcript to develop initial codes. The initial codes were reviewed and consolidated to produce a codebook that the researchers used to code the remaining transcripts. Each transcript was independently and iteratively coded by at least 2 of the researchers, with new codes added as needed. We grouped similar codes into domains on the basis of the interview guide and created new categories as needed, then examined the codes for overarching themes. The researchers met throughout the process to discuss coding, resolve disagreements, and identify exemplar quotes for major themes. We conducted a subgroup analysis of the focus group to identify differences between the modes of data collection. NVivo qualitative software (QSR International) was used to organize the data.

## Results

The full sample consisted of 16 women and 8 men, and the median age was 65 years (range, 48–82 y) (Table 2). Approximately 71% of participants had some college education, and 33% were married. Mean self-rated health was 3.5 (SD, 1.1), and arthritis interference was 5.2 (SD, 2.5). For the MHLC Form B, the Internal scale ranked highest at 4.4 (SD, 1.2), followed by Powerful Others at 3.5 (SD, 1.1) and Chance at 2.7 (SD, 1.0) (Table 1). For the MHLC Arthritis-Specific Form C, the Doctor scale had the highest mean score (4.4; SD, 1.0), followed by Internal (3.9; SD, 1.4) then Other People (2.8; SD, 1.1). We did not find significant differences between focus group and in-depth interview participants on any measures.

**Table 2:** Sociodemographic and Health-Related Characteristics of Older American Indians With Arthritis, Oregon, 2013

Characteristic	Overall Sample (N = 24)
**Age, y, median (range)**	65 (48–82)
**Sex, n (%)**	
Male	8 (33)
Female	16 (67)
**Education, n (%)**	
<High school diploma	2 (8)
High school or general educational development	5 (21)
Some college	12 (50)
College degree	5 (21)
**Marital status, n (%)**	
Married	8 (33)
Single	7 (29)
Divorced	6 (25)
Widowed	3 (13)
**Self-rated health^a^, mean (SD)**	3.5 (1.1)
**Arthritis interference scale^b^, mean (SD)**	5.2 (2.5)

Abbreviation: SD, standard deviation.

a Range, 1–6; higher scores indicate greater self-reported health.

b Range, 1–10; higher scores indicate greater self-reported interference from arthritis.

### Qualitative findings

The qualitative findings and representative quotes are organized by the domains outlined in the interview guide (Table 3). Major themes were generally consistent across focus groups and interviews; however, focus group participants reported being more physically active and more thoroughly discussed barriers to accessing medical care.

**Table 3:** Qualitative Themes Identified During Focus Groups and Interviews With Older American Indians Discussing Health, Arthritis, and Physical Activity Programs, Oregon, 2013

Domain	Sample Quotations
**Domain 1: Meaning of Health**	
The meaning of health is informed by cultural teachings passed down from elders and family	• “We had traditional meals (when we were raised) like. . . Indian roots. . . . That’s what (my grandparents) taught you, was to eat healthy foods and not . . . the stuff they have now.” (Female, I1) • “I was raised . . . with my uncles. They were there to give you good advice. . . . Always some way to take care of your body.” (Male participant, FG1)
Health is holistic	• “(Health is) taking care of your body physically, spiritually, emotionally, honoring your food.” (Female participant, I7)
Living a healthy lifestyle is central to health	• “(Health) means if I want to live or die, I’ve got to stick to my diet and do my exercise because . . . being healthy is going to keep me alive.” (Male participant, I15)
Health is the ability to move, to be active, and to take care of self and others	• “(Health is) where you can be more active at cooking . . . taking care of yourself. And take care of children and able to work.” (Female participant, I5) • “(Health is) to be able to get up every day, get out of bed, feed myself, and dress myself.” (Female participant, I11)
Health is freedom from pain and illness	• “(Health is) getting up without having to take . . . the 8 pills I take and not wait the hour for it to kick in.” (Male participant, I12)
The ability to live a healthy lifestyle has changed over time and affects the community	• “We lived off the land. . . . My grandparents cooked. They canned. They had cellars. I’m talking the old lifestyle that we lived.” (Male participant, I15) • “We have youth that aren’t well and they could be if they have things to look forward to like knowing more about health. . . . Some kids don’t have that. They don’t have the guidance and they need somebody to show them that there are better things for your body.” (Female participant, I1)
**Domain 2: Living with arthritis**	
Description of arthritis pain ranges from minimal to severe	• “It doesn’t really bother me much . . . it’ll come and go.” (Female participant, I1) • “Literally, some days . . . I wake up crying because it hurts so badly, and I just don’t want to do nothing.” (Female participant, I6)
Arthritis impacts ability to complete activities of daily living, to engage with family, and to do leisure activities	• “I can’t wash my own clothes” (Female participant, I2) • “It’s becoming less of a joy to go out and feed the horses.” (Male participant, I13) • “You don’t even want to hold a baby. You might drop it.” (Female participant, FG2) • “I was bead working before, making moccasins. I can’t do that now.” (Female participant, I3)
Confusion about what arthritis is and where it comes from	• “I don’t know if I have arthritis or osteoarthritis . . . . I don’t know the meaning of either one.” (Female participant, FG5)
**Coping strategies**	
Use of medication	• “With medication (my arthritis is) really good now” (Female participant, I2), • “(Medicine) didn’t really relieve my pain. It just took the edge off.” (Female participant, I3)
Culturally based strategies	• “What really helped me was going to a sweat lodge.” (Female participant, I6) • “We have medicinal, Native American plants that we use for arthritis.” (Female participant, FG4)
Distraction	• “(I) try to get (the pain) out of my head.” (Female participant, I5) • “It’s mind over matter.” (Male participant, I14)
Movement	• “Sometimes (moving) will hurt, but I notice it feels good afterwards. I won’t be stiff and I’ll get around better.” (Female participant, I1)
Access to medical care is challenging because of lack of local doctors, and the perception that Western doctors are not responsive	• “We just don’t have an arthritis doctor for us here on the reservation.” (Female participant, FG6) • “(Clinicians) don’t really take it to hear that you’re suffering. . . . That’s part of the reason why people don’t go to the clinic. . . . I think it’s natural for Native Americans to go back to old ways without any doctors and attention or somebody to help you out.” (Male participant, FG1)
**Domain 3: Barriers and facilitators to physical activity**	
**Barriers**	
Arthritis or other health conditions	• “It seems like after I had my surgery, I had a harder time walking.” (Female participant, I9) • “I can only do so much walking and then my stomach and back gets weak. . . . It was several years ago I had a hernia.” (Female participant, I3)
Lack of safe and accessible locations	• “Where you gonna walk without running into all these dogs along (the road)?” (Male participant, I16) • “Our native buildings should have our own gym.” (Female participant, I11)
Lack motivation or confidence that physical activity will help	• “I guess I just don’t want to be really active.” (Female participant, I4) • “Physical exertion and stretches. . . . I told myself they’re not helping. I feel like they’re making it worse.” (Male participant, I13)
**Facilitators**	
Focus on family	• “Someday there’s going to be grandkids and I have to be chasing . . . them. So I need to love myself now.” (Female participant, I5)
Fear of getting worse	• “I just don’t wanna get real bad like my friend. . . . She could hardly get around.” (Female participant, I7)
Wanting to be healthy	• “I try to stay on top of it because you know in the long run it is gonna affect me and nobody else.” (Female participant, I8)
**Domain 4: Preferences for program design**	
Interest in learning about arthritis	• “I am very interested in learning . . . pain management through exercise, nutrition and proper care versus the medication side of it.” (Female participant, FG4)
Preferences for group or solitary physical activity	• “I’d rather do it on my own.” (Female participant, I1) • “I've been looking into going to something like that with more of a structured thing instead of just showing up and hoping someone comes.” (Female participant, I11)
Schedule group classes around other community activities; provide transportation	• “If you could (offer a program) at breakfast (at the senior center), right after it or before it, then you could do that all in one.” (Female participant, I5) • “My main (barrier to participating) is transportation because I don’t drive.” (Male participant, I12)
Recruit by encouraging community members, engaging youth, and by showcasing successful community members	• “The main thing is to encourage them. I know a lot of people that . . . need that ‘come on let’s be a partner,’ somebody to let them know ‘I’ll go with you.’” (Female participant, I1) • “(Try) to get youth involvement and family activities they can all do together. It’d be fun. Might get more participation. That seemed to work (on another occasion).” (Male participant, I6) • “(If you) get people out on the floor and they’re all looking like, ‘Oh, them are Indian guys. They are Indian women. If they can do it, I can do it, too.’” (Male participant, I15)

Abbreviation: I, interview; FG, focus group.

Participants attributed their definitions of health to cultural values taught to them via their upbringing, traditions, and oral teachings from elders and family members. Health was a holistic concept that involved the mind, body, spirit, and caring for others. Living a healthy lifestyle that emphasized all these dimensions was considered central to health. Most participants described their upbringing as “healthy” in that they ate native foods (eg, salmon, roots, and berries) and participated in cultural activities that were physically active (eg, collecting roots and berries, fishing, hiking, preparing sweat houses, and riding horses). Several participants were concerned that there was a shift away from community events that promoted social engagement, creating fewer opportunities for the older adults to engage with youth and to pass down teachings about health.

All participants included functional ability in their definition of health. Functional ability was described as “being active.” For example, one participant said, “being able to move every day, able to do something.” Similarly, health involved independence and the ability to care for oneself, which enabled individuals to care for others. Illness and pain were seen as barriers to being active and healthy. Despite drawing the connection between healthy lifestyles as a way to facilitate health, participants struggled to maintain healthy behaviors and attributed these difficulties to changes in their local environment, in which availability of fast foods has increased and opportunities for physical activity have decreased.

The effect of arthritis on participants’ lives ranged from minimal and sporadic to overpowering and constant. Arthritis pain affected participants’ ability to complete activities of daily living, engage with family, take part in leisure activities, and get around. Many participants reported barriers to accessing medical care for their arthritis because of lack of local specialists and high staff turnover at the local clinic. Additionally, focus group participants discussed that clinicians did not understand or care about their pain or treat them respectfully. Several participants were unsure what arthritis was and confused arthritis pain and its treatment with their other conditions, such as spinal injuries, shingles, neuropathy, diabetes, and asthma.

Mechanisms for coping with arthritis pain were active and passive strategies that included using prescription and over-the-counter medications, nonpharmaceutical approaches, and physical movement. For some, medications were helpful in reducing pain but others experienced only minimal relief. Although over half of participants reported taking medications for arthritis, many preferred nonmedical interventions for arthritis and a few completely avoided clinical care. Nonpharmaceutical pain management included using heat or ice and rest, and traditional tribal remedies, including making teas from local plants and using sweat lodges and mineral baths. Many participants reported using mental strategies such as distraction and ignoring the pain.

Participants reported that “moving” was beneficial to reducing arthritis pain, but when probed about types of movements, they answered using generalized statements such as “I move around so I won’t get stiff;” or “I stretch . . . my legs and arms.” Few participants noted that physical activity, though initially painful, helped with their arthritis symptoms. Although almost all of the focus group participants engaged in some physical activity, no interview participants engaged in physical activity on a regular basis. Overall, the most common type of physical activity was walking.

Barriers to physical activity included the following themes: health status and pain, environmental and community factors, and lack of motivation. Many participants reported that pain caused by arthritis, chronic conditions, or injuries interfered with their ability to be active. Environmental and community barriers included lack of transportation, unsafe walking locations, and the cost of joining an exercise facility. Personal motivation was identified as both a barrier and a facilitator to physical activity. Some participants discussed how by motivating themselves, they were able to overcome arthritis pain to be active. They cited previous examples of using self-motivation to do healthy behaviors — including walking and losing weight — but described motivation as dwindling or insufficient to maintain these behaviors. Consequently, more than half of the sample identified lack of motivation as a barrier to currently being physically active. Those who wanted to be active reported being motivated by family, wanting to be healthy, and wanting to avoid negative outcomes that they had witnessed family members and friends experience.

Many participants had questions about arthritis, its causes and treatments, and about safe ways to exercise. Despite seeking information, participants voiced mixed interest in participating in an ongoing educational and physical activity program. About half of the sample was interested in a group exercise class, but some said they would not participate at all. Those interested in a program raised concerns that it would be difficult to find a suitable location, the classes might not be well-attended, and reliable transportation would be an issue. Given the transportation difficulties, a twice- or thrice-a-week class was not preferred. Participants suggested that engaging younger adults with families and youth in a health and physical activity program would be beneficial for the community. Other suggestions included providing transportation and incentives to participate, and recruiting through word-of-mouth, through locals or the leader providing personal outreach and encouragement, and by sharing success stories of local people who had improved through a similar program.

## Discussion

Our study examined older American Indians’ perceptions of health, arthritis, and physical activity to aid in identifying or adapting a suitable evidence-based program for a rural tribal community. Arthritis posed barriers to health and healthy behaviors, including physical activity. Meanings of health were couched in participants’ native values, upbringing, and traditions. Participants discussed the importance of being functionally independent in relationship to caring for themselves and others and to participating in cultural activities. This finding corroborates those of other studies that found that rural lay definitions of health are highly contextual, informed by rural lifestyles and local traditions (16). Similar to other American Indian populations, our sample lacked knowledge about arthritis and physical activity (17). Yet, participants were aware that movement was important for alleviating stiffness and immobility. Furthermore, there was a clear interest in learning about nonpharmaceutical coping strategies from both physically active and inactive participants. Together, these findings suggest that a culturally relevant physical activity program is desirable in this community. However, participants also identified critical barriers that should be addressed before such a program could be successfully implemented in their community.

Our findings suggest that only a few of the CDC-recommended physical activity programs for arthritis might be adaptable for this community. From an implementation perspective, most of the CDC-recommended programs require special facilities (eg, fitness facility or pool) and license fees that would be prohibitive in rural communities (see reference no. 4 for a detailed description of CDC-approved physical activity programs for arthritis). All of the programs are group-based and require regular meetings over an extended period that may pose challenges given the transportation issues identified. A possible adaptation to improve implementation could involve a peer-to-peer program conducted in-home or via telephone instead of in a group setting. Telephone-based coaching programs have shown promising results in promoting healthy behaviors (18). Furthermore, peer education programs are as effective as professionally delivered programs and are a viable option in communities where resources are limited (19). They also provide added benefits of localized knowledge and social support that that can support the health of rural older American Indians (20). Although several peer-led evidence-based arthritis programs exist (4), none are designed for in-home delivery or have been tested for use in rural settings.

In addition to the feasibility issues, existing programs should be adapted to ensure that messages and activities align with the values, contexts, and traditions unique to this tribe. Furthermore, any adaptations must align with the programs’ theoretical basis. Theory-based programs are not only more effective than those without (21), but also provide a framework for explaining the link between program components and resulting behavioral changes. Adaptations that significantly drift from the program’s theory-based components may result in decreased effectiveness or other undesirable outcomes (22). Attitudes, beliefs, and values, for example, are culturally informed constructs that are common to many behavioral change theories (23). Understanding the target populations’ beliefs and values about the program topic has implications for program adaptations. In our study, for example, the value that health is holistic indicates that a program focused solely on physical activity and arthritis might be too limited, and information about other aspects of healthy lifestyles, including nutrition, social relationships, and spiritual well-being, should be included to maximize relevance.

The participants in this study also emphasized the importance of health as a mechanism for caring for family members and for participating in social and cultural activities. They reflected on the teachings about health from their elders, and discussed their desire to engage with the youth in their community. This emphasis on the importance of social relationships has been reflected among other minority groups; other researchers suggest that common Western-based theoretical constructs, such as self-efficacy, that form the theoretical basis for interventions do not sufficiently represent the importance of relationships among minority groups in influencing health behavior (24). Interventions whose components draw from Western-based constructs may not be easily adapted to minority populations. Instead, interventions that attend to local contexts and values must be developed and tested to improve effectiveness, relevance, and acceptability. 

Another construct in need of further attention is that of the health locus of control. Overall, participants had high Internal locus of control on the general health scale, a finding that has been linked to healthy lifestyles including regular exercise (25). On further examination, however, separating out Doctors from Powerful Others via the arthritis-specific scale evidenced that doctors are attributed a higher level of control, a finding that has elsewhere been associated with poor health (26). Given the desire of our participants to avoid medical treatments and medications for arthritis, this finding is potentially confusing. One reason for this disconnect may lie in the qualitative findings that participants have difficulties accessing local and culturally sensitive health providers. O’Hea et al hypothesized that restricted access to health information could cause individuals to view their control of health as directly related to their ability to access health professionals (27). That is, people living in rural settings where transportation and access to health information is difficult may perceive that they are unable to take active control of their health given the difficulties in accessing resources. Developing and increasing accessibility to culturally appropriate information about arthritis self-management strategies has the potential to empower individuals to better manage their health.

Limitations to this study were the small sample size and the self-selection of participants, which should restrict generalizations of our findings to other American Indian groups. We had difficulty recruiting participants to focus groups, probably because of the transportation barriers identified by participants. Another limitation is that social desirability bias, especially in the focus group, may have affected responses by inflating reports of physical activity. Finally, the Cronbach α for the Other People subscale for Arthritis-Specific Form C was notably low, which suggests it had either poor internal consistency or too few items (only 3). Although the MHLC has been validated among American Indians, there are 566 distinct federally recognized tribes (28), each with its own values and traditions. Future studies are needed to further validate Form C among American Indians and to more thoroughly examine the applicability and implications of the health locus of control construct across tribes.

A tailored arthritis program that is relevant to local and cultural lifestyles, values, and beliefs of older, rural American Indians is needed to empower individuals to effectively manage their arthritis symptoms and reduce reliance on passive coping strategies. Adapting programs to improve implementation aspects for rural settings, such as delivering telephone or in-home programs, and incorporating culturally based values, such as integrating other aspects of health that acknowledge the holistic and social dimensions of health, are 2 potential considerations. A low-cost intervention strategy for older adults with arthritis that provides benefits of improved physical functioning and decreasing disability will be a significant contribution to mitigating the effect of arthritis among older American Indians.

## References

[1] Bolen J , Schieb L , Hootman JM , Helmick CG , Theis K , Murphy LB , Differences in the prevalence and severity of arthritis among racial/ethnic groups in the United States, National Health Interview Survey, 2002, 2003, and 2006. Prev Chronic Dis 2010;7(3):A64. Accessed December 1, 2015. 20394703 PMC2879996

[2] Hochberg MC , Altman RD , April KT , Benkhalti M , Guyatt G , McGowan J , American College of Rheumatology 2012 recommendations for the use of nonpharmacologic and pharmacologic therapies in osteoarthritis of the hand, hip, and knee. Arthritis Care Res (Hoboken) 2012;64(4):465–74. 10.1002/acr.21596 22563589

[3] Wilcox S , Castro C , King AC , Housemann R , Brownson RC . Determinants of leisure time physical activity in rural compared with urban older and ethnically diverse women in the United States. J Epidemiol Community Health 2000;54(9):667–72. 10.1136/jech.54.9.667 10942445 PMC1731735

[4] Centers for Disease Control and Prevention. Arthritis appropriate physical activity and self-management education interventions: a compendium of implementation information 2012. http://www.cdc.gov/arthritis/interventions/marketing-support/compendium/docs/pdf/compendium-2012.pdf. Accessed March 24, 2016.

[5] National Research Council (US) Committee on Population. Demography of American Indian elders: social, economic, and health status. In: Sandefur G, Rindfuss R, Cohen B, editors. Changing numbers, changing needs: American Indian demography and public health. Washington (DC): National Academies Press; 1996. p. 218–34. 25121291

[6] Wang-Schweig M , Kviz FJ , Altfeld SJ , Miller AM , Miller BA . Building a conceptual framework to culturally adapt health promotion and prevention programs at the deep structural level. Health Promot Pract 2014;15(4):575–84. 10.1177/1524839913518176 24396122 PMC4184993

[7] Leventhal H , Benyamini Y , Shafer C . Lay beliefs about health and illness. In: Ayers S, Baum A, McManus C, Newman S, Wallston KA, Weinman J, et al., editors. Cambridge handbook of psychology, health and medicine. New York (NY): Cambridge University; 2007. p. 124-7.

[8] National Indian Council on Aging. American Indian elderly: a national profile. Albuquerque (NM): Cordova Printino; 1981. http://files.eric.ed.gov/fulltext/ED219190.pdf. Accessed March 24, 2016.

[9] Ware JE , Kosinski M , Dewey JE , Gandek B . How to score and interpret single-item health status measures: a manual for users of the SF-8 health survey (with a supplement on the SF-6 health survey). Boston (MA): QualityMetric, Inc; 2001.

[10] Von Korff M , Ormel J , Keefe FJ , Dworkin SF . Grading the severity of chronic pain. Pain 1992;50(2):133–49. 10.1016/0304-3959(92)90154-4 1408309

[11] Armitage CJ , Norman P , Conner M . Can the Theory of Planned Behaviour mediate the effects of age, gender and multidimensional health locus of control? Br J Health Psychol 2002;7(Part 3):299–316. 10.1348/135910702760213698 12614502

[12] Wallston KA . The validity of the multidimensional health locus of control scales. J Health Psychol 2005;10(5):623–31. 10.1177/1359105305055304 16033784

[13] Egan JT , Leonardson G , Best LG , Welty T , Calhoun D , Beals J . Multidimensional health locus of control in American Indians: the strong heart study. Ethn Dis 2009;19(3):338–44. 19769018 PMC6097879

[14] Montague RB , Manson SM . Health locus of control and its assessment in older American Indians: National Technical Reports Library; 1997. https://ntrl.ntis.gov/NTRL/dashboard/searchResults/titleDetail/PB2003100233.xhtml. Accessed December 8, 2015.

[15] Hsieh HF , Shannon SE . Three approaches to qualitative content analysis. Qual Health Res 2005;15(9):1277–88. 10.1177/1049732305276687 16204405

[16] Goins RT , Spencer SM , Williams K . Lay meanings of health among rural older adults in Appalachia. J Rural Health 2011;27(1):13–20. 10.1111/j.1748-0361.2010.00315.x 21204968

[17] Mathews AE , Laditka SB , Laditka JN , Wilcox S , Corwin SJ , Liu R , Older adults’ perceived physical activity enablers and barriers: a multicultural perspective. J Aging Phys Act 2010;18(2):119–40. 20440026 10.1123/japa.18.2.119

[18] O’Hara BJ , Bauman AE , Eakin EG , King L , Haas M , Allman-Farinelli M , Evaluation framework for translational research: case study of Australia’s get healthy information and coaching service. Health Promot Pract 2013;14(3):380–9. 10.1177/1524839912456024 22982704

[19] Cohen JL , Sauter SV , deVellis RF , deVellis BM . Evaluation of arthritis self-management courses led by laypersons and by professionals. Arthritis Rheum 1986;29(3):388–93. 10.1002/art.1780290312 3964314

[20] Conte KP , Schure MB , Goins RT . Correlates of social support in older American Indians: the Native Elder Care Study. Aging Ment Health 2015;19(9):835–43. 10.1080/13607863.2014.967171 25322933 PMC5338610

[21] Noar SM , Zimmerman RS . Health Behavior Theory and cumulative knowledge regarding health behaviors: are we moving in the right direction? Health Educ Res 2005;20(3):275–90. 10.1093/her/cyg113 15632099

[22] Durlak JA , DuPre EP . Implementation matters: a review of research on the influence of implementation on program outcomes and the factors affecting implementation. Am J Community Psychol 2008;41(3-4):327–50. 10.1007/s10464-008-9165-0 18322790

[23] Flay BR , Snyder F , Petraitis J . The theory of triadic influence. In: DiClemente RJ, Kegler MC, Crosby RA, editors. Emerging theories in health promotion practice and research. 2nd edition. New York (NY): Jossey-Bass; 2009. p. 451–510.

[24] Pasick RJ , Burke NJ , Barker JC , Joseph G , Bird JA , Otero-Sabogal R , Behavioral theory in a diverse society: like a compass on Mars. Health Educ Behav 2009;36(5, Suppl):11S–35S. 10.1177/1090198109338917 19805789 PMC2921832

[25] Cobb-Clark DA , Kassenboehmer SC , Schurer S . Healthy habits: the connection between diet, exercise, and locus of control. J Econ Behav Organ 2013;98:1–28. 10.1016/j.jebo.2013.10.011

[26] Zautra AJ , Burleson MH , Smith CA , Blalock SJ , Wallston KA , DeVellis RF , Arthritis and perceptions of quality of life: an examination of positive and negative affect in rheumatoid arthritis patients. Health Psychol 1995;14(5):399–408. 10.1037/0278-6133.14.5.399 7498110

[27] O’Hea EL , Bodenlos JS , Moon S , Grothe KB , Brantley PJ . The multidimensional health locus of control scales: testing the factorial structure in sample of African American medical patients. Ethn Dis 2009;19(2):192–8. 19537232

[28] Bureau of Indian Affairs. Indian entities recognized and eligible to receive services from the United States Bureau of Indian Affairs. Fed Regist 2015;80(9):1942–8. https://www.loc.gov/catdir/cpso/biaind.pdf. Accessed December 8, 2015.

